# Multimorbidity patterns are associated with postoperative delirium in older patients undergoing non-cardiac surgery: an observational study

**DOI:** 10.3389/fmed.2026.1742763

**Published:** 2026-02-16

**Authors:** Xiao-Yi Hu, Di Fan, Xue-Yan Guo, Jian-Jun Yang, Mu-Huo Ji, Han-Wen Gu

**Affiliations:** 1 Department of Anesthesiology, Pain and Perioperative Medicine, The First Affiliated Hospital of Zhengzhou University, Zhengzhou, China; 2 Department of Anesthesiology, The Second Affiliated Hospital of Nanjing Medical University, Nanjing, China

**Keywords:** frailty, latent class analysis, machine learning, multimorbidity, postoperative delirium

## Abstract

**Background:**

Multimorbidity is associated with adverse outcomes among older adult surgical patients, yet its role in postoperative delirium (POD) remains unclear. In the present study, we hypothesized that distinct pattern of multimorbidity is associated with increased incidence of POD.

**Methods:**

From January 2024 to December 2024, 819 older adult patients were recruited at the Second Affiliated Hospital of Nanjing Medical University. Latent class analysis was used to identify patient subgroups based on disease composition. Mediation effect analysis explored the relationship between subgroups, Edmonton frail scale (EFS), and cognitive performance. Multinomial logistic regression model was employed to predict the subgroup to which patients with different diseases belong.

**Results:**

Three clinically distinct multimorbidity subgroups were identified. Significant differences in EFS, mini-mental state examination (MMSE), and POD were observed among subgroups (*p* < 0.05). After adjustment for age and MMSE, we found that subgroup 2 mediated the occurrence of POD through frailty [Indirect effect = 0.043; (95%CI = 0.019 ~ 0.070)]. Multinomial logistic regression model demonstrated good predictive power for subgroups, with AUROC scores as follows: subgroup 1 = 0.993, subgroup 2 = 0.977, and subgroup 3 = 0.990. The AUPRC scores were also strong, with subgroup 1 = 0.995, subgroup 2 = 0.886, and subgroup 3 = 0.974.

**Conclusion:**

We identified a specific pattern of multimorbidities significantly associated with frailty, cognitive impairment, and POD risk. The high-risk subgroup’s effect on POD was partially mediated by frailty. Multinomial logistic regression model accurately predicted subgroup membership, offering a potential tool for preoperative risk stratification.

## 1 Introduction

Postoperative delirium (POD) remains a significant and common complication among older surgical patients, associated with prolonged hospitalization, functional decline, and higher mortality rates (1, 2). While advanced age and preexisting cognitive impairment are established risk factors, the role of multimorbidity—defined as the coexistence of two or more diseases—remains inadequately characterized despite its high prevalence in this population (3, 4).

Older adults increasingly present with complex and heterogeneous multimorbidity patterns, yet current clinical approaches largely remain focused on single diseases, often overlooking the synergistic and cumulative effects of concurrent conditions on postoperative outcomes. Current evidence suggests that specific clusters of diseases, rather than simply the number of multimorbidities, may more accurately capture vulnerability to adverse events (5). The etiology of multimorbidity is multifactorial, involving behavioral patterns, living environment, genetic factors, and social-psychological influences, whose interactions may evolve nonlinearly over time and lead to highly variable clinical phenotypes (6, 7). Conventional multimorbidity indices, though useful in some contexts, often fail to account for these nuanced patterns and their differential impact on postoperative recovery.

This study aims to address the salient knowledge gap by pursuing a dual objective: First, we will identify distinct multimorbidity subgroups among older surgical patients using latent class analysis (LCA), which is increasingly employed to reveal unobserved groups with distinct clinical characteristics from heterogeneous datasets (8–10). Second, to bridge the gap between subgroup identification and clinical application, we developed and validated a predictive model capable of classifying individual patients into these subgroups based on the presence or absence of specific clinical diseases. We hypothesize that specific multimorbidity subgroups are associated with an increased risk of POD and that these subgroups can be accurately predicted using routinely available clinical data. By elucidating the relationship between multimorbidity patterns and POD and providing a practical tool for preoperative risk stratification, this study seeks to support targeted interventions and ultimately reduce the incidence of POD in older surgical populations.

## 2 Methods

### 2.1 Patients’ recruitment

This prospective, observational study was conducted in accordance with the STROBE reporting guidelines and received approval from the Ethics Committee of the Second Affiliated Hospital of Nanjing Medical University (Approval No. 2024-KY-007-01) (11). The study was registered at the Chinese Clinical Trial Registry (ChiCTR2400080808). All participants provided informed consent prior to recruitment, either directly or through a legally authorized representative.

The inclusion criteria were: (1) Voluntary participation with signed informed consent approved by the Ethics Committee; (2) Age ≥ 65 years; (3) Expected hospital stay of at least 3 days; and (4) Undergoing elective, non-cardiac surgery. The exclusion criteria were: (1) Inability to communicate due to coma, severe dementia, or language impairment; (2) Severe abnormalities of heart, brain, kidney or liver; (3) History of major trauma or major surgery within the past year; and (4) Dysfunction in vital organs.

### 2.2 Definition of multimorbidity and POD

Diseases were categorized using the International Classification of Diseases, 10th Revision (ICD-10) diagnostic standards. For example, liver diseases included conditions such as liver cysts, liver mass lesions, and cirrhosis. Pulmonary diseases encompassed lung nodules, bronchiectasis, and bronchial asthma. Connective tissue diseases were classified to include conditions like rheumatoid arthritis and systemic lupus erythematosus, while immune diseases included ulcerative colitis, psoriasis, and Sjögren’s syndrome. Common diseases with high prevalence, such as hypertension, diabetes, and coronary heart disease, were documented under their respective ICD-10 codes.

The 3D Confusion Assessment Method (3D-CAM) was used to assess POD twice daily during the first 3 days postoperatively (12). The assessment criteria included: (1) changes in the level of consciousness; (2) acute fluctuations in mental status; (3) disorganized thinking, and (4) inattention. A diagnosis of POD was established when both criteria (1) and (2) were met, along with either (3) or (4), or both. All research staff conducting the 3D-CAM assessments underwent a standardized training session to ensure consistent interpretation and application of the instrument.

### 2.3 Statistical analyses

Student’s *t* test, Mann–Whitney U-test, or Chi-square were used, as appropriate, for comparisons between two groups. For multiple group comparisons, one-way ANOVA or the Kruskal-Wallis test was used, with Bonferroni correction applied to adjust for multiple comparisons. Continuous variables are presented as means ± standard deviations for normally distributed data, or as medians with interquartile ranges (IQR) for non-normally distributed data. Categorical variables are expressed as numbers (percentages). A *p*-value of less than 0.05 was considered statistically significant.

### 2.4 LCA

LCA was used to identify multimorbid subgroups, incorporating various disease classification characteristics. LCA is based on the assumption that unobserved (“latent”) subgroups exist within the study cohort. We followed the methodological approach outlined by Zhou et al. to identify and validate these subgroups (13). The optimal number of subgroups was determined by selecting the model with the lowest values for Bayesian Information Criteria (BIC) and Akaike Information Criteria (AIC), ensuring that each subgroup comprised at least 5% of the study population. For our sample of 819 individuals, this corresponded to a minimum subgroup size of 41 samples (14).

### 2.5 Sample size calculation

The required sample size for the multivariable logistic regression analysis was determined based on the Events Per Variable (EPV) rule, as recommended by Wang et al. (15). Given that the analysis included six predictors—three latent classes (represented by two dummy variables) and four confounding factors (EFS, MMSE, gender, and age)—a minimum of 10 events per variable was required, totaling 60 POD events. With an anticipated POD incidence rate of 20%, the initial sample size was calculated as follows (16): Initial sample size = Number of events/Event rate = 600/0.2 = 300 participants. To account for potential variability within the latent classes identified by LCA, a design effect (DEEF) of 2 was applied, resulting in an adjusted sample size of 600 participants (17). Furthermore, considering an estimated 20% data ineligibility or loss, the final required sample size was adjusted to ensure sufficient power: Final sample size = 600/(1–0.20) = 750 participants. Therefore, a total of 750 participants were deemed necessary to achieve adequate statistical power (80%) for detecting significant associations between the latent classes and POD while controlling for the specified confounding variables.

### 2.6 Network visualization

Network analysis was used to visualize the complex relationships among different diseases, offering insights into associations that might otherwise be difficult to discern. In this approach, each node in the network represented distinct diseases, while the edges connecting nodes quantified the relationships using relative risk (RR). Disease-disease associations were quantified using Risk Ratios (RR) derived from 2 × 2 contingency tables, calculated with the epi.2by2 function (R package epiR). RR was chosen over the Odds Ratio for its more direct clinical interpretability as a measure of relative risk in our study population. The width of the edges indicated the strength of the RR between diseases, with thicker edges representing stronger associations. The size of each node reflected the prevalence of the corresponding disease within the study population.

### 2.7 Mediation analysis

Mediation analysis was used to break down the relationship between the multimorbid subgroup (“X”) and postoperative delirium (POD) (“Y”) into direct and indirect effects. The indirect effect is facilitated by a mediator (“M”), which converts the effect of “X” on “M” (path “a”) into the effect of “M” on “Y” (path “b”), with the magnitude of the mediating effect represented by the product of “a” and “b.” We hypothesized that frailty (“M”) lies on the causal pathway (X → M → Y) between the multimorbid subgroup (“X”) and POD (“Y”). For a variable to qualify as a mediator, the following conditions must be met (18): (1) X must significantly predict Y; (2) X must significantly predict M; (3) M must significantly predict Y; (4) The mediating effect (“ab”) must be statistically significant; (5) The existence of multipathic subgroups and frailty theoretically fulfills the minimal conditions for causal mediation. Multimorbid subgroups were treated as independent variables, with age and MMSE scores included as covariates in generalized linear regression to model POD. In a second analysis, multimorbid subgroups served as the independent variable, with age as a covariate, to model MMSE. The mediation package in R software was used to model structural equations and estimate both direct and indirect effects. Statistical significance for each effect size was determined through simulation (1,000 iterations), yielding bias-corrected 95% confidence intervals (CI).

### 2.8 Prediction for multimorbidity subgroups

Multinomial logistic regression model was constructed to predict the identified multimorbidity subgroups, using the multinom() function from the nnet package in R software. The dataset was divided into a training set (70%) and a validation set (30%). To address class imbalance and improve model generalizability, we employed a 5-fold cross-validation approach combined with the synthetic minority over-sampling technique (SMOTE). The SMOTE algorithm was applied exclusively to the training folds during cross-validation to augment minority class samples while preserving the original distribution of the validation folds. Model performance was assessed on the validation set using the area under the receiver operating characteristic curve (AUROC) and the area under the precision-recall curve (AUPRC). An area under the curve (AUC) value closer to 1 indicates superior model performance, while an AUC of 0.5 suggests a performance equivalent to random guessing (19).

## 3 Results

### 3.1 Baseline characteristics

The cohort demographics were detailed in Supplementary Table S1 in the supplementary materials. Among the 819 older adult patients, 82.1% had two or more medical conditions, with a prevalence of POD at 17.9%. Compared with those without POD, patients in the POD group were older, had higher Edmonton Frail Scale (EFS) scores, increased rates of neurological disorders, anemia, and endocrine diseases, and lower MMSE scores (*p* < 0.05).

**Figure 1:**
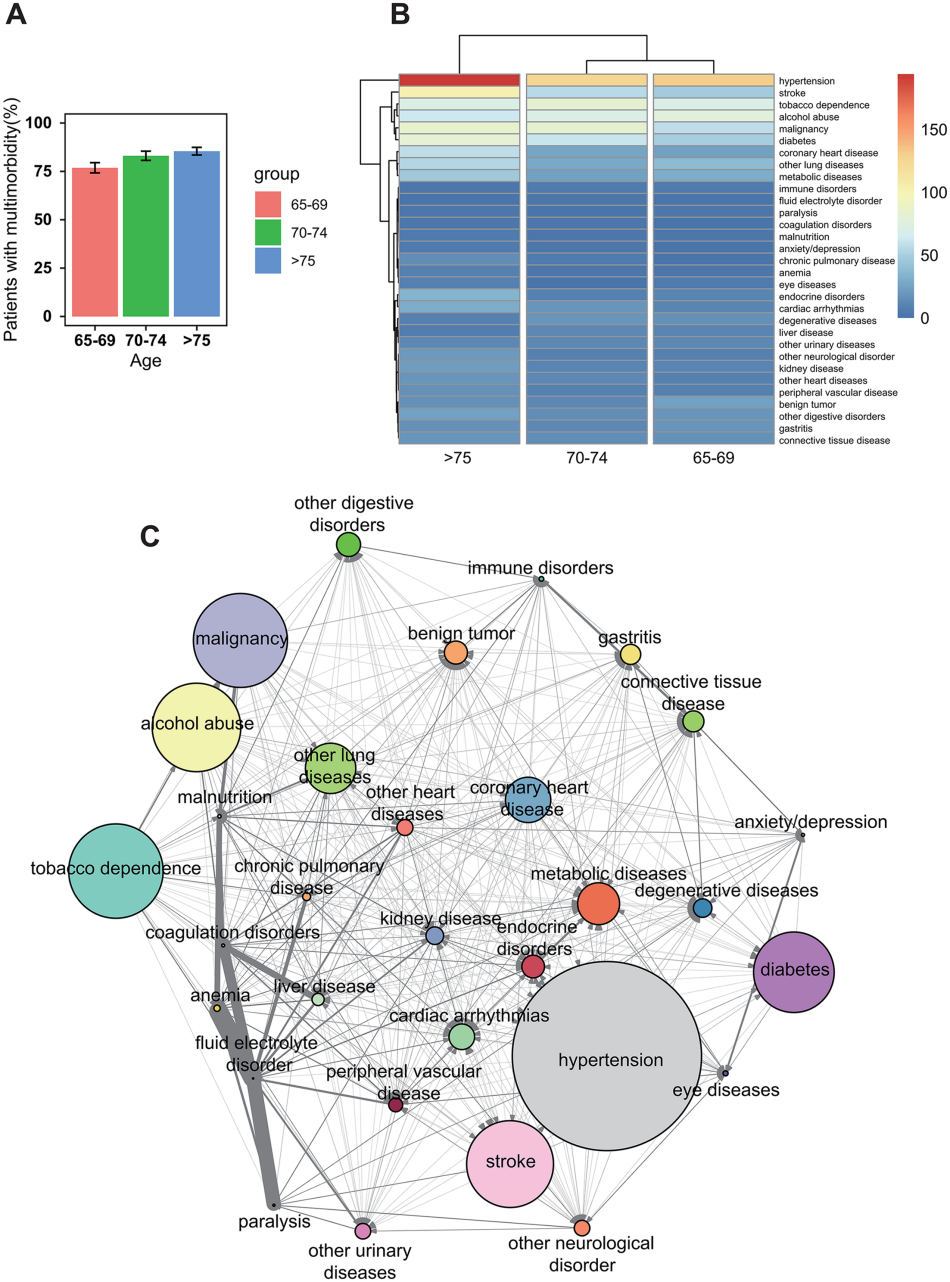
The heterogeneous morbidity profile of the patients. **(A)** The percentage of multimorbid patients gradually increases with age (mean ± standard deviation). **(B)** The distribution of different diseases across various age groups; the heatmap illustrates the heterogeneous composition of different diseases; the color code (on the right) represents the number of cases for each disease. **(C)** Network visualization analysis in the study cohort reveals the correlations between diseases; node size represents disease prevalence, while edge width indicates the relative risk between diseases.

As depicted in Figure 1A, the prevalence of multimorbidity increased with age. Figure 1B illustrated distinct patterns in disease prevalence across age groups: (1) increased incidence with age (e.g., hypertension, cerebral infarction, coronary heart disease); (2) low overall prevalence (e.g., anxiety/depression, malnutrition, hemiplegia); (3) a U-shaped incidence trend with age (e.g., benign tumors, metabolic diseases, and digestive diseases). Network visualization in Figure 1C demonstrated frequent associations among malignancy, malnutrition, anemia, liver disease, coagulation dysfunction, water and electrolyte imbalances, and hemiplegia, supporting the hypothesis of potential subgroups within the cohort. This tightly linked cluster provides visual evidence of a distinct disease complex, supporting the existence of clinically relevant multimorbidity subgroups. The network analysis reinforces the validity of the subgroups identified via LCA.

### 3.2 Identification of distinct multimorbidity subgroups

**Figure 2:**
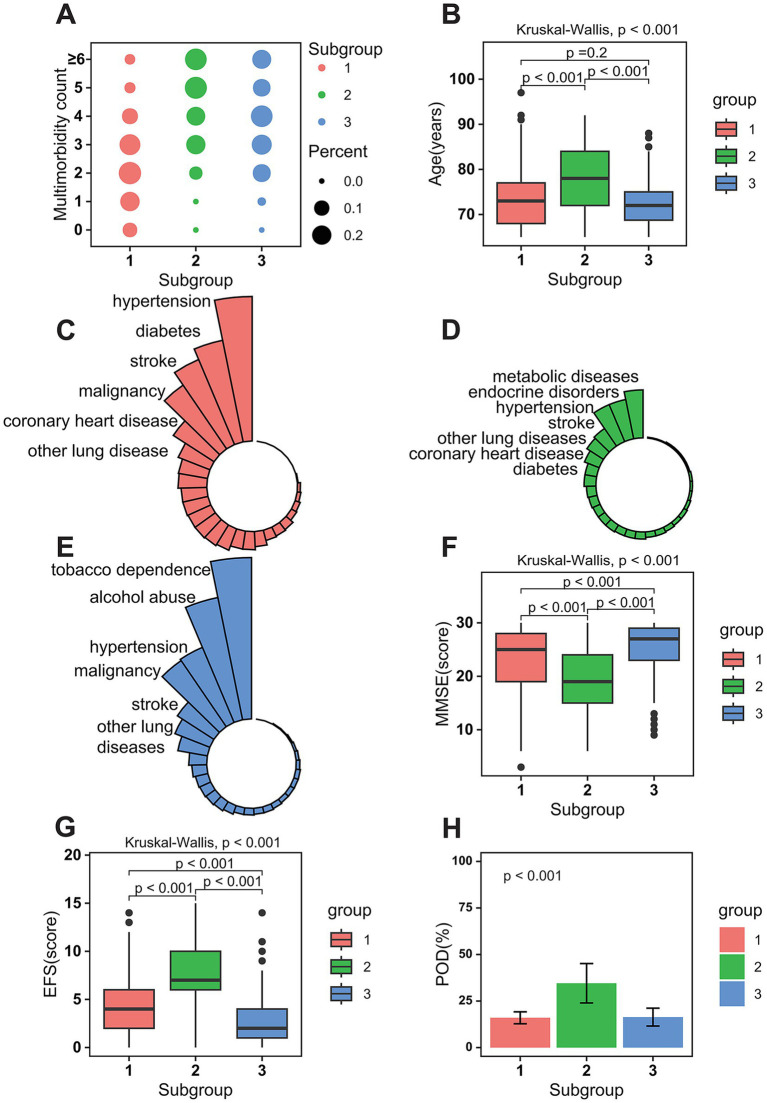
Characteristics of the three identified subgroups in the study cohort. **(A)** Bubble plot summary of multimorbidity incidence counts for each subgroup. **(B)** Distribution of age among different subgroups. **(C–E)** Distribution of different diseases among subgroup 1 to 3; **(F–H)** Distribution of MMSE scores, EFS scores, and POD occurrence among different subgroups; MMSE, mini-mental state examination (range: 0–30); EFS, Edmonton Frail Scale (range: 0–18); POD, postoperative delirium. A higher EFS score indicates a greater level of frailty; a lower MMSE score indicates greater cognitive impairment, with lower scores reflecting more severe deficits in areas such as memory, attention, and orientation.

Based on the relationships among various diseases, we hypothesized the existence of multimorbidity subgroups characterized by distinct demographics and disease compositions. Using LCA, we identified three distinct patient subgroups (Supplementary Table S2 and Figures 2A–E). Subgroup 1 was primarily characterized by a high burden of cardiometabolic and cerebrovascular conditions, including hypertension, diabetes mellitus, and cerebral infarction. Subgroup 2 represented the oldest patients and was predominantly defined by metabolic and endocrine disorders, such as metabolic diseases and endocrine diseases, alongside other multimorbidities. Subgroup 3 was most notably distinguished by behavioral health factors, specifically tobacco dependence and alcohol abuse, co-occurring with other conditions. Detailed comparisons among the subgroups were available in Supplementary Table S3.

### 3.3 Frailty as a mediator in POD among multimorbidity subgroups

**Figure 3:**
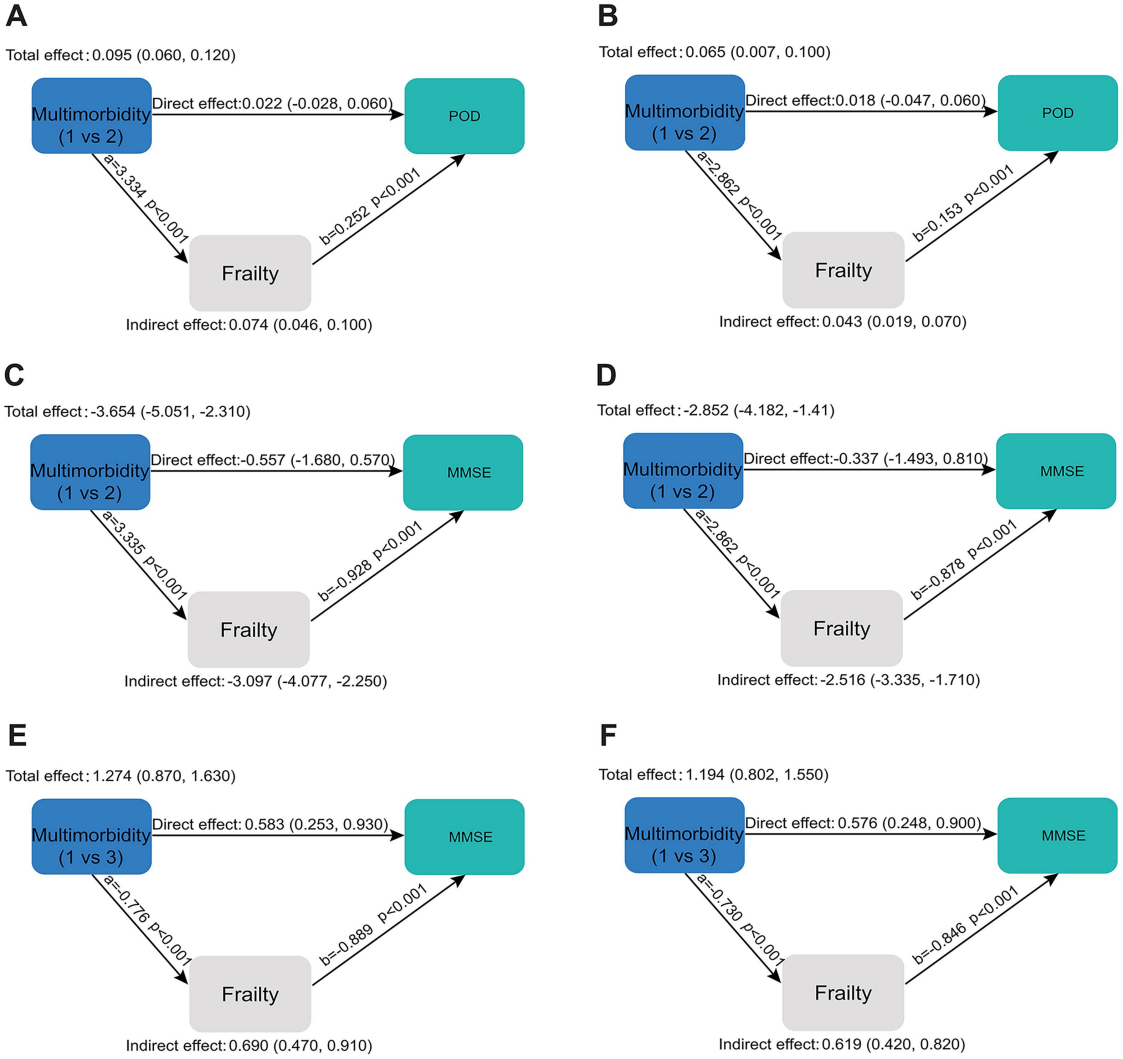
Standardized path coefficients of the mediation model, both unadjusted and adjusted. **(A,B)** Multimorbidity subgroup (1 vs. 2) as the independent variable, considering frailty as the mediator variable, and POD as the dependent variable; summary of unadjusted **(A)** and adjusted **(B)** standardized path coefficients of the mediation model; **(C–F)** multimorbidity subgroups (1 vs. 2 and 1 vs. 3) as the independent variables, considering frailty as the mediator variable, and MMSE as the dependent variable; summary of unadjusted **(C,E)** and adjusted **(D,F)** standardized path coefficients of the mediation model. MMSE, mini-mental state examination (range: 0–30); EFS, Edmonton Frail Scale (range: 0–18); POD, postoperative delirium. A higher EFS score indicates a greater level of frailty; a lower MMSE score indicates greater cognitive impairment, with lower scores reflecting more severe deficits in areas such as memory, attention, and orientation.

We assessed differences in adverse health outcomes, including frailty, cognitive impairment, and POD, across the multimorbid subgroups (Figures 2F–H). Statistically significant differences were observed in MMSE scores, EFS scores, and POD occurrence between the subgroups (*p* < 0.001). These findings informed the hypothesis for the subsequent mediation analysis. The results of the mediation analysis were presented in Supplementary Table S4, and Figure 3 illustrated the normalized path coefficients, both adjusted and unadjusted, for the mediation model. Comparing subgroups 1 and 2, Figure 3A illustrated a mediating effect of frailty on POD (0.074; 95% CI: 0.046 ~ 0.100). After adjusting for relevant confounders (Figure 3B), the mediating effect between frailty and POD remained significant (0.043; 95% CI: 0.019 ~ 0.070). Additionally, comparisons between subgroups 1 and 2 (Figures 3C,D) and between subgroups 1 and 3 (Figures 3E,F) also revealed significant mediating effects of frailty on MMSE scores, which persisted even after adjusting for confounders.

### 3.4 Prediction of the multimorbidity subgroup

**Figure 4:**
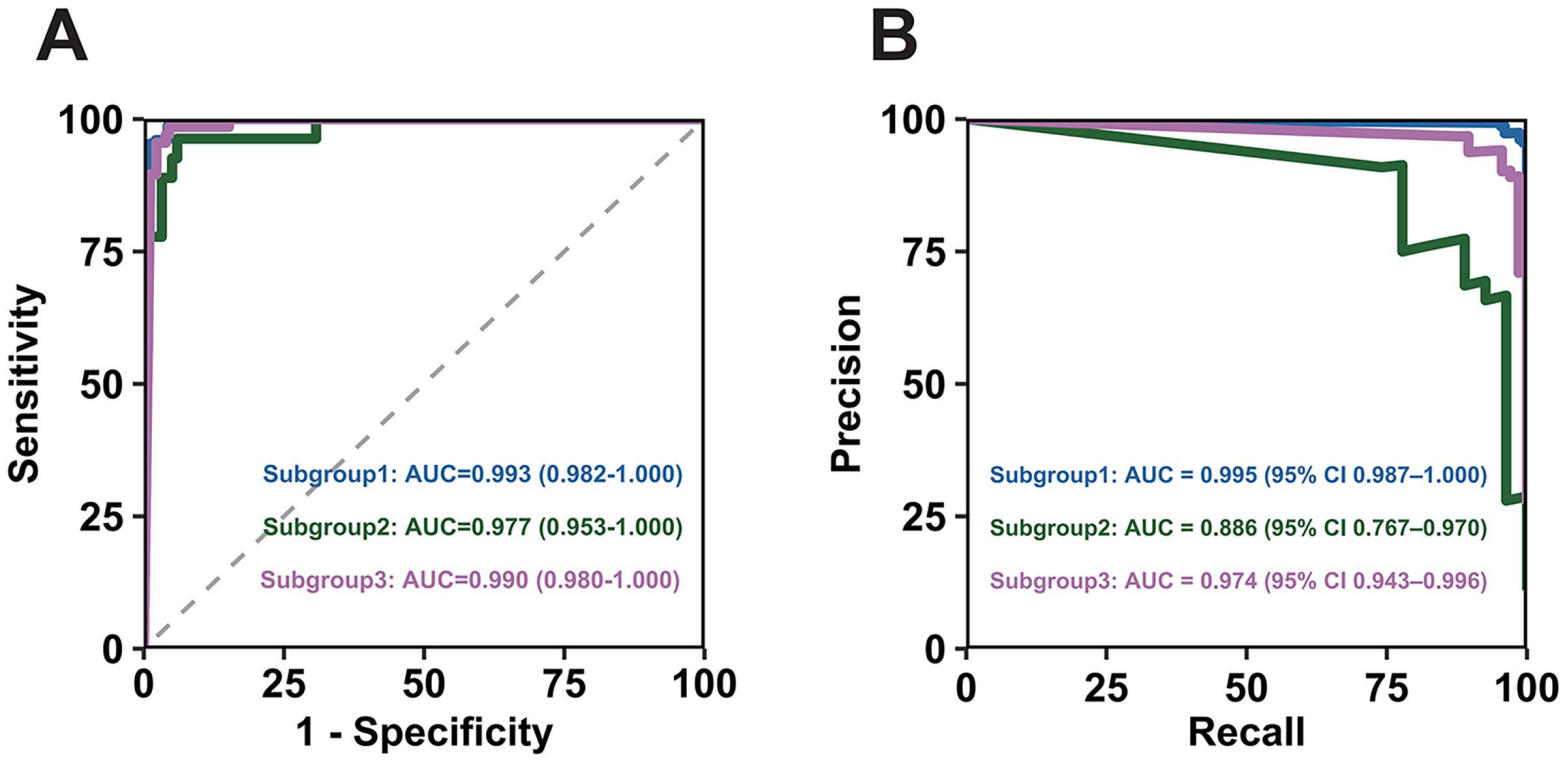
A series of performance metrics in the multinomial logistic regression model. **(A)** Area under the receiver operating characteristic curve for different multimorbidity subgroups. **(B)** Area under the precision-recall curve for different multimorbidity subgroups.

After identifying clinically significant multimorbidity subgroups, we developed a multinomial logistic regression model to predict subgroup membership for each patient. The model demonstrated excellent predictive performance across different subgroups [Subgroup 1: AUROC = 0.993; Subgroup 2: AUROC = 0.977; Subgroup 3: AUROC = 0.9990 and Subgroup 1: AUPRC = 0.995; Subgroup 2: AUPRC = 0.886; Subgroup 3: AUPRC = 0.974]. For detailed information, please refer to Figure 4 and Supplementary Table S5.

## 4 Discussion

In this prospective cohort study, we identified three distinct multimorbidity subgroups among older surgical patients and demonstrated their varying risks for adverse outcomes. Crucially, we developed a multinomial logistic regression model capable of accurately predicting these subgroups, providing a feasible tool for clinical translation. These findings advocate for a paradigm shift from a single-disease model to a multidimensional, subgroup-based framework in perioperative care.

Consistent with previous research, we observed that multimorbidity prevalence increases with age (20). The multimorbidity subgroups identified through LCA exhibited clinically meaningful profiles: Subgroup 1 was characterized by a relatively younger age and a high prevalence of common cardiovascular conditions, including hypertension (17.92%), diabetes mellitus (12.85%), and cerebral infarction (11.44%). Although patients in this subgroup presented with multiple comorbidities, this disease cluster is frequently observed in clinical practice. Subgroup 3 was marked by distinct behavioral health features, dominated by tobacco dependence (22.83%) and alcohol abuse (17.65%), accompanied by hypertension (11.52%) and malignancy (11.31%). Most notably, Subgroup 2 demonstrated the most clinically relevant profile: these patients were the oldest and exhibited a core pattern of metabolic diseases (15.33%) and endocrine disorders (12.81%), along with hypertension (6.78%) and cerebral infarction (6.28%). Crucially, this specific disease combination not only defines a unique metabolic-endocrine phenotype but is also associated with the highest incidence of POD. The clinical implication of this finding is that Subgroup 2 represents a high-risk population warranting particular attention—older adults characterized by metabolic and endocrine dysregulation. Preoperative identification of patients with this clinical profile is essential, as it enables the implementation of targeted interventions such as metabolic and endocrine optimization, comprehensive geriatric assessment, and multimodal delirium prevention strategies, thereby potentially improving surgical outcomes.

The relationship between aging, frailty, and cognitive impairment is well-documented. As physiological function declines with age, frailty emerges when the cellular repair mechanisms fail to maintain homeostasis in response to stress (21). Research consistently shows that frailty, characterized by decreased physiological reserves and increased vulnerability to stressors, is a major risk factor for POD, especially in older surgical patients (22). This vulnerability is likely due to the compromised ability of frail patients to maintain homeostasis during postoperative recovery, making them more susceptible to cognitive impairment. Our study aligns with previous findings that link frailty to increased POD incidence, while emphasizing the role of specific multimorbidity patterns. Notably, we found that the influence of multimorbidity subgroup 2 on cognitive impairment was mediated by frailty, even after adjusting for confounders. The identified subgroups suggest that the combination of certain diseases—such as metabolic and endocrine disorders—interacts with frailty, creating a compounded risk. These interactions highlight the complexity of managing older adult patients, as multiple health conditions amplify vulnerability to adverse outcomes. This finding aligns with the observed higher proportion of older patients with multiple morbidities in subgroup 2, supporting the need for targeted interventions in this population. Previous studies have also highlighted the role of aging and comorbidities in increasing POD risk, even outside the context of cardiac surgery (23, 24). Therefore, perioperative care should include effective frailty screening and multidisciplinary strategies—such as nutritional support, psychological preparation, and physical exercise—to improve frailty in patients with multimorbidity.

After identifying subgroups associated with adverse health outcomes, a key limitation of our study is the lack of standardized criteria for classifying new patients into these subgroups, restricting clinical adoption. It is important to emphasize that the multinomial logistic regression model was applied not for causal inference, but specifically to address this translational challenge (25). While the LCA identified the subgroups, the multinomial logistic regression model served as a pragmatic predictive classifier, enabling the assignment of future patients to the most probable subgroup based on their clinical disease profiles (26, 27). The high predictive accuracy (as indicated by the AUROC and AUPRC values) demonstrates that the complex patterns of diseases defining each LCA-derived subgroup can be reliably mapped using a computational model. This justifies its application by showing that the subgroups are not merely statistical constructs but are clinically recognizable and predictable from routine data. The value of this approach lies in its potential for risk stratification—flagging high-risk individuals (e.g., those likely belonging to subgroup 2) preoperatively based on their multimorbidities. This facilitates targeted, pre-emptive interventions for patients at greatest risk of adverse outcomes like POD, moving a step closer to personalized perioperative care.

The study has limitations inherent in observational studies. First, the single-center prospective study focused on a small sample of older adult patients undergoing non-cardiac surgery, the findings may have limited generalizability. Large, independent, multicenter clinical trials are necessary to confirm the findings. Second, the study lacks laboratory data, potentially overlooking important factors (28). Third, for classification and statistical power considerations, we combined diseases of varying clinical severity under broader organ-system categories (e.g., grouping liver cysts with cirrhosis, lung nodules with asthma). While this approach facilitated the LCA, it may mask the independent impact of specific, severe diseases on POD. Fourth, a key consideration is the complex interrelationship between frailty, cognitive function, nutritional status, and comorbidity. These factors are deeply intertwined and likely exhibit bidirectional relationships. While we positioned frailty as a mediator based on *a priori* clinical reasoning and temporality, it may also share confounding relationships with other syndromes. Our mediation analysis should therefore be interpreted as exploring a statistically defined and clinically plausible pathway, rather than providing definitive causal proof. The intricate interplay between these variables remains a challenge for observational studies and warrants future investigation with longitudinal designs capable of better establishing temporal sequence. Fifth, our study diagnosed POD as a binary outcome and did not differentiate between clinical subtypes (e.g., hyperactive, hypoactive, or mixed). Future investigations into whether specific multimorbidity patterns are associated with distinct delirium subtypes could provide further pathophysiological and management insights. Lastly, we relied on a single frailty measure, which, despite its clinical utility, may not capture the full spectrum of frailty as comprehensively as a multi-component assessment would (29). Additionally, although POD was assessed twice daily using standardized tools, the fluctuating nature of POD means that some episodes may have been undetected between assessment intervals.

## 5 Conclusion

Our study identified three distinct multimorbidity subgroups among older surgical patients, among which the subgroup characterized by metabolic-endocrine diseases was associated with the highest risk of postoperative delirium, partially mediated through frailty. Furthermore, the multinomial logistic regression model demonstrated high accuracy in predicting these subgroups based on clinical disease profiles, providing a feasible tool for pre-operative risk stratification and targeted interventions.

## Data Availability

The original contributions presented in the study are included in the article/supplementary material, further inquiries can be directed to the corresponding authors.
