# Supplementary material for: Multimorbidity patterns are associated with postoperative delirium in older patients undergoing non-cardiac surgery: an observational study
Source: Front Med (Lausanne). 2026 Feb 16;13:1742763. doi: 10.3389/fmed.2026.1742763 (PMC12950787; doi:10.3389/fmed.2026.1742763)
Supplement: Supplementary file 1 [file Data_Sheet_1.docx]

Table S1. Comparison of the general characteristics between the two groups of patients.

| Variables | Total (n = 819) | Non-POD (n = 672) | POD (n = 147) | p |
| --- | --- | --- | --- | --- |
| Gender, n (%) |  |  |  | 0.236 |
| Female | 313 (38) | 250 (37) | 63 (43) |  |
| Male | 506 (62) | 422 (63) | 84 (57) |  |
| Age, Median (Q1,Q3) | 73 (69, 77) | 72 (68, 76) | 76 (71, 83) | < 0.001 |
| Tobacco dependence, n (%) | 225 (27) | 186 (28) | 39 (27) | 0.857 |
| Alcohol abuse, n (%) | 211 (26) | 173 (26) | 38 (26) | 1.000 |
| Malignancy, n (%) | 224 (27) | 177 (26) | 47 (32) | 0.198 |
| Other heart diseases, n (%) | 38 (5) | 31 (5) | 7 (5) | 1.000 |
| Coronary heart disease, n (%) | 108 (13) | 82 (12) | 26 (18) | 0.100 |
| Chronic pulmonary disease, n (%) | 19 (2) | 13 (2) | 6 (4) | 0.130 |
| Other lung diseases, n (%) | 120 (15) | 98 (15) | 22 (15) | 1.000 |
| Stroke, n (%) | 207 (25) | 163 (24) | 44 (30) | 0.184 |
| Hypertension, n (%) | 451 (55) | 373 (56) | 78 (53) | 0.654 |
| Diabetes, n (%) | 192 (23) | 160 (24) | 32 (22) | 0.673 |
| Liver diseases, n (%) | 29 (4) | 27 (4) | 2 (1) | 0.183 |
| Gastritis, n (%) | 48 (6) | 42 (6) | 6 (4) | 0.412 |
| Other digestive disorders, n (%) | 57 (7) | 47 (7) | 10 (7) | 1.000 |
| Other neurological disorder, n (%) | 37 (5) | 23 (3) | 14 (10) | 0.003 |
| Kidney diseases, n (%) | 42 (5) | 34 (5) | 8 (5) | 1.000 |
| Other urinary diseases, n (%) | 37 (5) | 31 (5) | 6 (4) | 0.951 |
| Connective tissue diseases, n (%) | 50 (6) | 44 (7) | 6 (4) | 0.347 |
| Anemia, n (%) | 15 (2) | 7 (1) | 8 (5) | 0.002 |
| Coagulation disorders, n (%) | 7 (1) | 6 (1) | 1 (1) | 1.000 |
| Paralysis, n (%) | 5 (1) | 2 (0) | 3 (2) | 0.043 |
| Peripheral vascular diseases, n (%) | 33 (4) | 28 (4) | 5 (3) | 0.845 |
| Endocrine disorders, n (%) | 54 (7) | 33 (5) | 21 (14) | < 0.001 |
| Metabolic diseases, n (%) | 100 (12) | 83 (12) | 17 (12) | 0.901 |
| Benign tumor, n (%) | 55 (7) | 40 (6) | 15 (10) | 0.092 |
| Malnutrition, n (%) | 8 (1) | 7 (1) | 1 (1) | 1.000 |
| Fluid electrolyte disorder, n (%) | 3 (0) | 1 (0) | 2 (1) | 0.085 |
| Anxiety/depression, n (%) | 7 (1) | 7 (1) | 0 (0) | 0.363 |
| Cardiac arrhythmias, n (%) | 61 (7) | 47 (7) | 14 (10) | 0.376 |
| Immune disorders  , n (%) | 11 (1) | 11 (2) | 0 (0) | 0.229 |
| Degenerative diseases, n (%) | 44 (5) | 39 (6) | 5 (3) | 0.333 |
| Eye diseases, n (%) | 12 (1) | 9 (1) | 3 (2) | 0.460 |
| EFS, Median (Q1,Q3) | 4 (2, 6) | 3 (2, 6) | 6 (3, 11) | < 0.001 |
| MMSE, Median (Q1,Q3) | 25 (20, 28) | 26 (21, 28) | 19 (15, 25) | < 0.001 |

Notes: A higher EFS score indicates a greater level of frailty; A lower MMSE score indicates greater cognitive impairment, with lower scores reflecting more severe deficits in areas such as memory, attention, and orientation; Abbreviation: EFS, edmonton frail scale (range: 0-18); MMSE,mini-mental state examination (range: 0-30);

Table S2. Fit indices of latent class analysis on multimorbidity status

| Number of latent classes | Log  likelihood | Df | AIC | BIC | BLRT  (p value) |
| --- | --- | --- | --- | --- | --- |
| 1 | -6579.931 | 33 | 13225.86 | 13381.23 |  |
| 2 | -6362.581 | 68 | 12861.16 | 13212.38 | <0.001 |
| 3 | -6260.726 | 103 | 12727.45 | 13181.31 | <0.001 |
| 4 | -6208.729 | 138 | 12904.17 | 13553.89 | <0.001 |
| 5 | -6579.931 | 173 | 13303.42 | 14117.92 | 1.000 |

Notes: The BLRT p-values for 2, 3, and 4 latent classes are statistically significant (p < 0.001), suggesting improved model fit with increasing numbers of latent classes. However, the 5-class model shows a BLRT p-value of 1.000, indicating no improvement in fit compared to the 4-class model. Based on these results, the 3-class model is considered the most optimal for the data. Abbreviation: AIC, Akaike information criteria; BIC, Bayesian information criteria; BLRT, Bootstrap's likelihood ratio test;

Table S3. Comparison of the general characteristics between the three subgroups.

| Variables | Total  (n = 819) | Subgroup 1  (n = 506) | Subgroup 2  (n = 81) | Subgroup 3  (n = 232) | p |
| --- | --- | --- | --- | --- | --- |
| POD, n (%) | 147 (18) | 81 (16) | 28 (35) | 38 (16) | < 0.001 |
| Gender, n (%) |  |  |  |  | < 0.001 |
| Female | 313 (38) | 239 (47) | 28 (35) | 46 (20) |  |
| Male | 506 (62) | 267 (53) | 53 (65) | 186 (80) |  |
| Age, Median (Q1,Q3) | 73 (69, 77) | 73 (68, 77) | 78 (72, 84) | 72 (68.75, 75) | < 0.001 |
| Tobacco dependence, n (%) | 225 (27) | 0 (0) | 9 (11) | 216 (93) | < 0.001 |
| Alcohol abuse, n (%) | 211 (26) | 39 (8) | 5 (6) | 167 (72) | < 0.001 |
| Malignancy, n (%) | 224 (27) | 102 (20) | 15 (19) | 107 (46) | < 0.001 |
| Other heart diseases, n (%) | 38 (5) | 12 (2) | 12 (15) | 14 (6) | < 0.001 |
| Coronary heart disease, n (%) | 108 (13) | 64 (13) | 20 (25) | 24 (10) | 0.004 |
| Chronic pulmonary disease, n (%) | 19 (2) | 0 (0) | 10 (12) | 9 (4) | < 0.001 |
| Other lung diseases, n (%) | 120 (15) | 45 (9) | 25 (31) | 50 (22) | < 0.001 |
| Stroke, n (%) | 207 (25) | 122 (24) | 27 (33) | 58 (25) | 0.206 |
| Hypertension, n (%) | 451 (55) | 291 (58) | 51 (63) | 109 (47) | 0.009 |
| Diabetes, n (%) | 192 (23) | 137 (27) | 15 (19) | 40 (17) | 0.008 |
| Liver disease, n (%) | 29 (4) | 13 (3) | 4 (5) | 12 (5) | 0.128 |
| Gastritis, n (%) | 48 (6) | 32 (6) | 9 (11) | 7 (3) | 0.020 |
| Other digestive disorders, n (%) | 57 (7) | 34 (7) | 7 (9) | 16 (7) | 0.818 |
| Other neurological disorder, n (%) | 37 (5) | 24 (5) | 7 (9) | 6 (3) | 0.078 |
| Kidney disease, n (%) | 42 (5) | 24 (5) | 10 (12) | 8 (3) | 0.014 |
| Other urinary diseases, n (%) | 37 (5) | 20 (4) | 2 (2) | 15 (6) | 0.235 |
| Connective tissue disease, n (%) | 50 (6) | 38 (8) | 6 (7) | 6 (3) | 0.021 |
| Anemia, n (%) | 15 (2) | 1 (0) | 9 (11) | 5 (2) | < 0.001 |
| Coagulation disorders, n (%) | 7 (1) | 0 (0) | 3 (4) | 4 (2) | < 0.001 |
| Paralysis, n (%) | 5 (1) | 4 (1) | 1 (1) | 0 (0) | 0.204 |
| Peripheral vascular disease, n (%) | 33 (4) | 14 (3) | 10 (12) | 9 (4) | 0.001 |
| Endocrine disorders, n (%) | 54 (7) | 0 (0) | 51 (63) | 3 (1) | < 0.001 |
| Metabolic diseases, n (%) | 100 (12) | 33 (7) | 61 (75) | 6 (3) | < 0.001 |
| Benign tumor, n (%) | 55 (7) | 30 (6) | 5 (6) | 20 (9) | 0.390 |
| Malnutrition, n (%) | 8 (1) | 0 (0) | 2 (2) | 6 (3) | < 0.001 |
| Fluid electrolyte disorder, n (%) | 3 (0) | 0 (0) | 3 (4) | 0 (0) | < 0.001 |
| Anxiety/depression, n (%) | 7 (1) | 5 (1) | 1 (1) | 1 (0) | 0.500 |
| Cardiac arrhythmias, n (%) | 61 (7) | 33 (7) | 11 (14) | 17 (7) | 0.080 |
| Immune disorders  , n (%) | 11 (1) | 7 (1) | 2 (2) | 2 (1) | 0.462 |
| Degenerative diseases, n (%) | 44 (5) | 34 (7) | 2 (2) | 8 (3) | 0.109 |
| Eye diseases, n (%) | 12 (1) | 8 (2) | 3 (4) | 1 (0) | 0.098 |
| EFS, Median (Q1,Q3) | 4 (2, 6) | 4 (2, 6) | 7 (6, 10) | 2 (1, 4) | < 0.001 |
| MMSE, Median (Q1,Q3) | 25 (20, 28) | 25 (19, 28) | 19 (15, 24) | 27 (23, 29) | < 0.001 |
| Number of multimorbidity, Median (Q1,Q3) | 3 (2, 4) | 2 (1, 3) | 5 (3, 6) | 4 (3, 5) | < 0.001 |

Abbreviation: POD, postoperative delirium; EFS, edmonton frail scale (range: 0-18); MMSE, mini-mental state examination (range: 0-30);

Table S4. Standardised mediation effect size of frailty.

| Model | Total effect (95% CI) | Direct effect (95% CI) | Indirect effect (95% CI) |
| --- | --- | --- | --- |
| POD |  |  |  |
| 1 vs 2 (unadjusted) | 0.095 (0.060, 0.120)* | 0.022 (-0.028, 0.060) | 0.074 (0.046, 0.100)* |
| 1 vs 3 (unadjusted) | 0.004 (-0.026, 0.030) | 0.025 (-0.002, 0.050) | -0.021 (-0.032, -0.010) |
| 1 vs 2 (adjusted) | 0.065 (0.007, 0.100)* | 0.018 (-0.047, 0.060) | 0.043 (0.019, 0.070)* |
| 1 vs 3 (adjusted) | 0.018 (-0.008, 0.040) | 0.029 (0.005, 0.050) | -0.011 (-0.021, -0.004) |
| MMSE |  |  |  |
| 1 vs 2 (unadjusted) | -3.654 (-5.051, -2.310)^#^ | -0.557 (-1.680, 0.570) | -3.097 (-4.077, -2.250)^#^ |
| 1 vs 3 (unadjusted) | 1.274 (0.870, 1.630)^#^ | 0.583 (0.253, 0.930) | 0.690 (0.470, 0.910)^#^ |
| 1 vs 2 (adjusted) | -2.852 (-4.182, -1.410)^#^ | -0.337 (-1.493, 0.810) | -2.516 (-3.335, -1.710)^#^ |
| 1 vs 3 (adjusted) | 1.194 (0.802, 1.550)^#^ | 0.576 (0.248, 0.900) | 0.619 (0.420, 0.820)^#^ |

Notes: *Multimorbidity status as the independent variable, EFS score as the mediating variable, POD as the outcome variable. The adjusted confounders included age, MMSE. ^#^Multimorbidity status as the independent variable, EFS score as the mediating variable, MMSE score as the outcome variable. The adjusted confounders included age. Abbreviation: EFS, edmonton frail scale (range: 0-18); MMSE,mini-mental state examination (range: 0-30); POD postoperative delirium; *^#^, p < 0.05;

Table S5. Comparison of general features between the training and testing sets.

| Variables | Total  (n = 819) | Testing  (n = 245) | Training  (n = 574) | p |
| --- | --- | --- | --- | --- |
| Gender, n (%) |  |  |  | 0.516 |
| Female | 313 (38) | 89 (36) | 224 (39) |  |
| Male | 506 (62) | 156 (64) | 350 (61) |  |
| Age, Median (Q1,Q3) | 73 (69, 77) | 73 (68, 77) | 73 (69, 77) | 0.549 |
| Tobacco dependence, n (%) | 225 (27) | 63 (26) | 162 (28) | 0.515 |
| Alcohol abuse, n (%) | 211 (26) | 64 (26) | 147 (26) | 0.947 |
| Malignancy, n (%) | 224 (27) | 61 (25) | 163 (28) | 0.346 |
| Other heart diseases, n (%) | 38 (5) | 11 (4) | 27 (5) | 1.000 |
| Coronary heart disease, n (%) | 108 (13) | 30 (12) | 78 (14) | 0.683 |
| Chronic pulmonary disease, n (%) | 19 (2) | 5 (2) | 14 (2) | 0.926 |
| Other lung diseases, n (%) | 120 (15) | 35 (14) | 85 (15) | 0.932 |
| Stroke, n (%) | 207 (25) | 65 (27) | 142 (25) | 0.651 |
| Hypertension, n (%) | 451 (55) | 138 (56) | 313 (55) | 0.692 |
| Diabetes, n (%) | 192 (23) | 48 (20) | 144 (25) | 0.107 |
| Liver diseases, n (%) | 29 (4) | 6 (2) | 23 (4) | 0.369 |
| Gastritis, n (%) | 48 (6) | 12 (5) | 36 (6) | 0.546 |
| Other digestive disorders, n (%) | 57 (7) | 23 (9) | 34 (6) | 0.102 |
| Other neurological disorders, n (%) | 37 (5) | 9 (4) | 28 (5) | 0.564 |
| Kidney diseases, n (%) | 42 (5) | 15 (6) | 27 (5) | 0.503 |
| Other urinary diseases, n (%) | 37 (5) | 12 (5) | 25 (4) | 0.874 |
| Connective tissue diseases, n (%) | 50 (6) | 18 (7) | 32 (6) | 0.418 |
| Anemia, n (%) | 15 (2) | 4 (2) | 11 (2) | 1.000 |
| Coagulation disorders, n (%) | 7 (1) | 2 (1) | 5 (1) | 1.000 |
| Paralysis, n (%) | 5 (1) | 3 (1) | 2 (0) | 0.161 |
| Peripheral vascular diseases, n (%) | 33 (4) | 9 (4) | 24 (4) | 0.885 |
| Endocrine disorders, n (%) | 54 (7) | 19 (8) | 35 (6) | 0.471 |
| Metabolic diseases, n (%) | 100 (12) | 37 (15) | 63 (11) | 0.125 |
| Benign tumor, n (%) | 55 (7) | 18 (7) | 37 (6) | 0.750 |
| Malnutrition, n (%) | 8 (1) | 2 (1) | 6 (1) | 1.000 |
| Fluid electrolyte disorder, n (%) | 3 (0) | 2 (1) | 1 (0) | 0.215 |
| Anxiety/depression, n (%) | 7 (1) | 1 (0) | 6 (1) | 0.681 |
| Cardiac arrhythmias, n (%) | 61 (7) | 21 (9) | 40 (7) | 0.513 |
| Immune disorders, n (%) | 11 (1) | 4 (2) | 7 (1) | 0.741 |
| Degenerative diseases, n (%) | 44 (5) | 12 (5) | 32 (6) | 0.823 |
| Eye diseases, n (%) | 12 (1) | 3 (1) | 9 (2) | 1.000 |
| Number of multimorbidity, Median (Q1,Q3) | 4 (3, 5) | 4 (3, 5) | 4 (3, 5) | 0.798 |
| Subgroup, n (%) |  |  |  | 0.751 |
| 1 | 506 (62) | 151 (62) | 355 (62) |  |
| 2 | 81 (10) | 27 (11) | 54 (9) |  |
| 3 | 232 (28) | 67 (27) | 165 (29) |  |
